# An approach to the diagnosis of lumbar disc herniation using deep learning models

**DOI:** 10.3389/fbioe.2023.1247112

**Published:** 2023-09-04

**Authors:** Ardha Ardea Prisilla, Yue Leon Guo, Yih-Kuen Jan, Chih-Yang Lin, Fu-Yu Lin, Ben-Yi Liau, Jen-Yung Tsai, Peter Ardhianto, Yori Pusparani, Chi-Wen Lung

**Affiliations:** ^1^ Department of Fashion Design, LaSalle College Jakarta, Jakarta, Indonesia; ^2^ Department of Digital Media Design, Asia University, Taichung, Taiwan; ^3^ Environmental and Occupational Medicine, College of Medicine, National Taiwan University (NTU) and NTU Hospital, Taipei, Taiwan; ^4^ Graduate Institute of Environmental and Occupational Health Sciences, College of Public Health, National Taiwan University, Taipei, Taiwan; ^5^ National Institute of Environmental Health Sciences, National Health Research Institutes, Miaoli, Taiwan; ^6^ Rehabilitation Engineering Lab, Department of Kinesiology and Community Health, University of Illinois at Urbana-Champaign, Urbana, IL, United States; ^7^ Department of Mechanical Engineering, National Central University, Taoyuan, Taiwan; ^8^ Department of Neurology, China Medical University Hospital, Taichung, Taiwan; ^9^ Department of Automatic Control Engineering, Feng Chia University, Taichung, Taiwan; ^10^ Department of Visual Communication Design, Soegijapranata Catholic University, Semarang, Indonesia; ^11^ Department of Visual Communication Design, Budi Luhur University, Jakarta, Indonesia; ^12^ Department of Creative Product Design, Asia University, Taichung, Taiwan

**Keywords:** augmentation, automatic detection, low back pain, MRI, YOLO models

## Abstract

**Background:** In magnetic resonance imaging (MRI), lumbar disc herniation (LDH) detection is challenging due to the various shapes, sizes, angles, and regions associated with bulges, protrusions, extrusions, and sequestrations. Lumbar abnormalities in MRI can be detected automatically by using deep learning methods. As deep learning models gain recognition, they may assist in diagnosing LDH with MRI images and provide initial interpretation in clinical settings. YOU ONLY LOOK ONCE (YOLO) model series are often used to train deep learning algorithms for real-time biomedical image detection and prediction. This study aims to confirm which YOLO models (YOLOv5, YOLOv6, and YOLOv7) perform well in detecting LDH in different regions of the lumbar intervertebral disc.

**Materials and methods:** The methodology involves several steps, including converting DICOM images to JPEG, reviewing and selecting MRI slices for labeling and augmentation using ROBOFLOW, and constructing YOLOv5x, YOLOv6, and YOLOv7 models based on the dataset. The training dataset was combined with the radiologist’s labeling and annotation, and then the deep learning models were trained using the training/validation dataset.

**Results:** Our result showed that the 550-dataset with augmentation (AUG) or without augmentation (non-AUG) in YOLOv5x generates satisfactory training performance in LDH detection. The AUG dataset overall performance provides slightly higher accuracy than the non-AUG. YOLOv5x showed the highest performance with 89.30% mAP compared to YOLOv6, and YOLOv7. Also, YOLOv5x in non-AUG dataset showed the balance LDH region detections in L2-L3, L3-L4, L4-L5, and L5-S1 with above 90%. And this illustrates the competitiveness of using non-AUG dataset to detect LDH.

**Conclusion:** Using YOLOv5x and the 550 augmented dataset, LDH can be detected with promising both in non-AUG and AUG dataset. By utilizing the most appropriate YOLO model, clinicians have a greater chance of diagnosing LDH early and preventing adverse effects for their patients.

## Highlight

1. The YOLOv5x in the dataset with augmentation (AUG) successfully performed the highest mAP compared to all YOLO models tested, which indicated the model with the highest performance model to detect lumbar disc herniation (LDH).
2. YOLOv5x without augmentation (non-AUG) dataset performed well in detecting LDH in L2-L3, L3-L4, L4-L5, and L5-S1 regions with values above 90%, which showed effectiveness in using a non-AUG dataset for training.
3. YOLOv5x showed the shortest training duration and the lightest weight compared to YOLOv6 and YOLOv7, indicating the most efficient LDH detection model.

## 1 Introduction

Lumbar disc herniation (LDH) is caused by the bulging or rupture of a spinal disc segment, misaligning its position, and irritating the nerve roots, which causes sciatica (Vialle et al., 2010; Amin et al., 2017). In particular, the lumbar vertebrae are more susceptible to misalignment since they support the body’s weight (Mushtaq et al., 2022). LDH occurs in 2%–3% of the worldwide population, interfering with everyday activities and productivity (Vialle et al., 2010). The cost of treating lumbar disc herniation in the United States with medications and surgery amounted to $4.0 billion in 2015 (Martin et al., 2019).

Four most commonly occurring forms of LDH include bulging, protrusion, extrusion, and sequestration (Gopalakrishnan et al., 2015). These forms are caused by a rupture of the fibrous layers of the annulus (the bony outer shell), which can cause a leak of the nucleus pulposus (soft inner core) and irritate adjacent nerve roots (Gopalakrishnan et al., 2015). An early diagnosis of LDH can assist in curing the disease in its earliest stages and protect the patient from harmful repercussions. One of the most used medical imaging techniques for diagnosing LDH is magnetic resonance imaging (MRI) (Choi et al., 2017). The use of MRI in diagnosing LDH has been widely adopted because of its ability to reveal the shape of intervertebral discs. Intervertebral discs with elliptical shapes are robust, whereas discs with abnormal shapes are deformed and flattened (Alomari et al., 2014; Chen et al., 2021).

However, clinicians must undergo extensive training to interpret and analyze MRI of LDH. Although well-trained medical professionals can analyze the MRI, the diagnosis results might be inconsistent (Alomari et al., 2014). Radiologists are reported to be biased in their interpretation of MRIs, with disagreement on a variation of bulging discs, which indicates the need for standardized mechanisms in MRI interpretations (Alomari et al., 2014). Researchers have demonstrated that MRI LDH can be detected automatically by using deep learning methods, which can increase radiology practice efficiency (Azimi et al., 2020). By using deep learning to interpret MRI images, the existing bias variability can be reduced, and diagnostic decisions can be standardized (Alomari et al., 2014). It has been shown that deep learning architectures can successfully address image recognition and classification accurately from MRI using automated learning features (Tsai et al., 2021). Consequently, researchers strive to improve performance results while designing different deep learning architectures (Azimi et al., 2020).

In the past, two-stage detectors deep learning models were used to classify and detect LDH in MRI. Alomari et al. (2014) proposed utilizing a coordinated active shape and a gradient vector flow active contour models to extract shape features for detecting LDH. Wang et al., proposed a two-stage detector fusion model that utilizes DenseNet and Inception-Resnet-V2 models to increase the number of image features and improve image recognition accuracy (Wang et al., 2020). Su et al., developed another two-stage detector model using ResNet-50 that consists of three fully connected networks based on a backbone network for feature extraction and performs classification tasks on lumbar MRI of LDH (Su et al., 2022).

Several studies adopted the single-stage detector YOU ONLY LOOK ONCE (YOLO) model to train deep learning algorithms for real-time biomedical image detection and prediction established on anchor base and intersection over union techniques (Tsai et al., 2021; Guinebert et al., 2022; Mushtaq et al., 2022). Our previous study tested only YOLOv3 in Darknet to detect LDH in MRI (Tsai et al., 2021). However, a variety of more recent YOLO models have been developed, including YOLOv5, YOLOv6, and YOLOv7 (Jocher, 2020; Li et al., 2022; Wang et al., 2022). Contrary to previous releases based on Darknet, these models are based on PyTorch, which is more typically used for computer vision and natural language processing (Jocher, 2020; Li et al., 2022; Wang et al., 2022).

YOLOv5 has been used in biomedical image detection with promising results (Mushtaq et al., 2022). According to Mushtaq et al., YOLOv5 performance has been reported to be higher in accuracy than YOLOv3 at identifying lumbar lordotic angles (LLA) and lumbosacral angles (LSA) (Mushtaq et al., 2022). However, YOLOv6 and YOLOv7 were published in June and July 2022 and remain relatively novel (Li et al., 2022; Wang et al., 2022). An evaluation of YOLOv5, YOLOv6, and YOLOv7 has been conducted to determine which model detects safety helmets the most effectively. YOLOv7 outperformed YOLOv5 and YOLOv6 in detecting safety helmets (Yung et al., 2022). However, further research is required to confirm YOLOv6 and YOLOv7 effectiveness compared to YOLOv5 in detecting biomedical images.

To the best of our knowledge, YOLOv5, YOLOv6, and YOLOv7 have not yet been evaluated for their ability to detect LDH in MRI, although the three models provide promising object detection performance. In YOLOv5, several features performed well on the validation set and were more efficient during interpretation (Jocher, 2020). YOLOv6 also outperforms YOLOv5 in detection accuracy and is more confident about its label in industrial applications (Li et al., 2022). YOLO7 is also reported to be more accurate in detecting Microsoft common objects in context (MS COCO) than previous YOLO models (Wang et al., 2022).

Our previous study has shown that YOLOv3 could detect LDH in MRI best with image augmentation (Tsai et al., 2021). However, since YOLOv5, YOLOv6, and YOLOv7 have improved image detection features, we hypothesized our study aims as follows:

•  Based on YOLO metrics performance, we would like to compare YOLOv5, YOLOv6, and YOLOv7 models for LDH detection to determine which model would provide the highest level of accuracy.
•  To assess further how well YOLO models perform, we would like to compare them with and without the augmentation dataset.
•  The three YOLO models will be used to determine the optimal training duration for clinical use.

An accurate model can assist clinicians in determining the LDH earlier in MRI images. Following this, we will discuss the materials and methods used for the proposed deep learning models, which include datasets and detailed methodologies. In addition, we will discuss the performance results of models which achieved the LDH detection results and explain them in greater detail.

## 2 Materials and methods

### 2.1 Image dataset

MRI images were derived from a publicly available dataset of lumbar spines by Sudirman et al. (2019a) (https://data.mendeley.com/datasets/k57fr854j2/2) based on an anonymized clinical study. The dataset was collected from patients at the Irbid Specialty Hospital in Jordan who reported symptomatic back pain between September 2015 and July 2016 (Al-Kafri et al., 2019; Sudirman et al., 2019a). The lumbar MRI images were stored in Digital Imaging and Communications in Medicine (DICOM) files. In order to ensure similar physiology for the lumbar spine, the MRI images were taken from patients at least 17 years of age (Al-Kafri et al., 2019). MRI images include T1 and T2 weighted images with sagittal and axial views. Most images have a resolution of 320x320 pixels with a precision of 12-bit per pixel (Sudirman et al., 2019a). We extracted the DICOM data and converted the images to JPEG by using the provided MATLAB code from the dataset source (Sudirman et al., 2019b).

**FIGURE 1:**
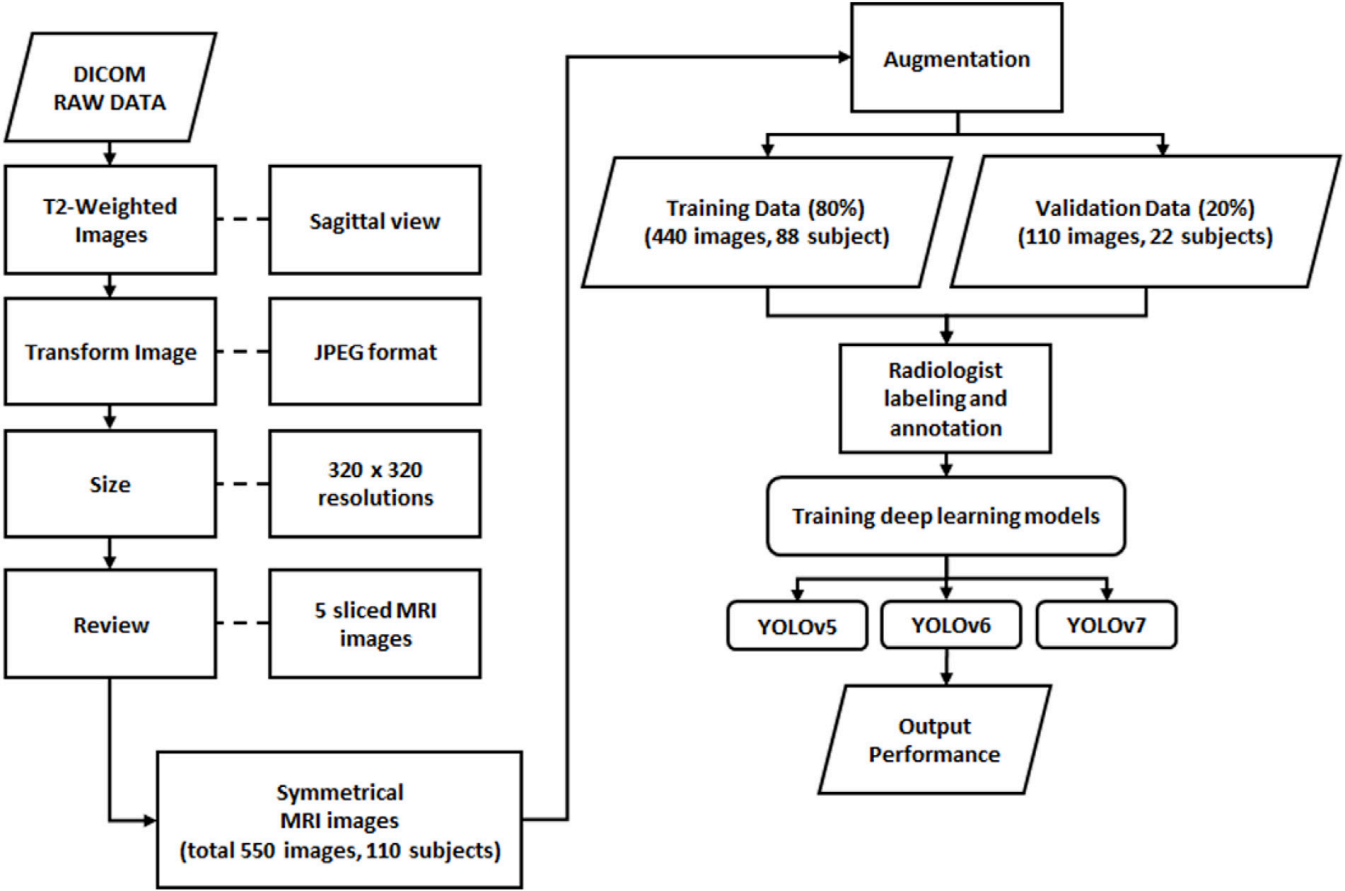
Process flow of data processing for object detection in deep learning.

Before deep learning model training, all images must be examined, standardized, and transformed into organized data (Tsai et al., 2021) (Figure 1). From the DICOM raw data, we used T2-weighted images to obtain better brightness and darkness features. A clinical trial has been conducted to demonstrate the acceptable equivalent of using T2-WI in sagittal to assess LDH (Alomari et al., 2014). Therefore, we used the T2-WI in the sagittal view in this study. In this study, a total of 550 images were used from 110 different subjects. The ground truth and MATLAB code for data extraction were also provided from the source (Sudirman et al., 2019b). We supervised the selection of subjects for this study based on the ground truth, which included only individuals with disc herniations between L1 and S1. We used five midsagittal slices for each subject, including the middle slice and two symmetrical slices on either side of the vertebral body, representing approximately the full transverse diameter of the vertebral body (Friska and Sudirman, 2021). We selected five slices that provided clear views of the lumbar region. The 550 images were divided into 80% training images (440 images from 88 subjects) and 20% validation images (110 images from 22 subjects). The sagittal view of upper and lower lumbar MRI has been used in a clinical trial for the detection and segmentation of lumbar MRI (Ghosh and Chaudhary, 2014). Hence in this study, we use the sagittal view as the initial identification of LDH. Further analysis was performed by combining the training and validation datasets with radiologists’ diagnosis records.

### 2.2 Object detection architecture

Object detection techniques have been widely utilized in many medical diagnostic applications, such as YOLO algorithms for detecting medical images in MRI (Tsai et al., 2021; Mushtaq et al., 2022). The YOLO framework uses a single-stage detection approach for real-time object recognition. Single-stage detectors are designed to detect objects using relatively simple architecture by focusing on all the spatial regions. It improves detection accuracy and reduces the time required for inferences (Jocher, 2020; Li et al., 2022; Wang et al., 2022). Advances in YOLO models over the years have resulted in different performance levels among the models. This study assesses the MRI lumbar image and determines the bounding box based on the performance of the YOLOv5, YOLOv6, and YOLOv7 models.

**FIGURE 2:**
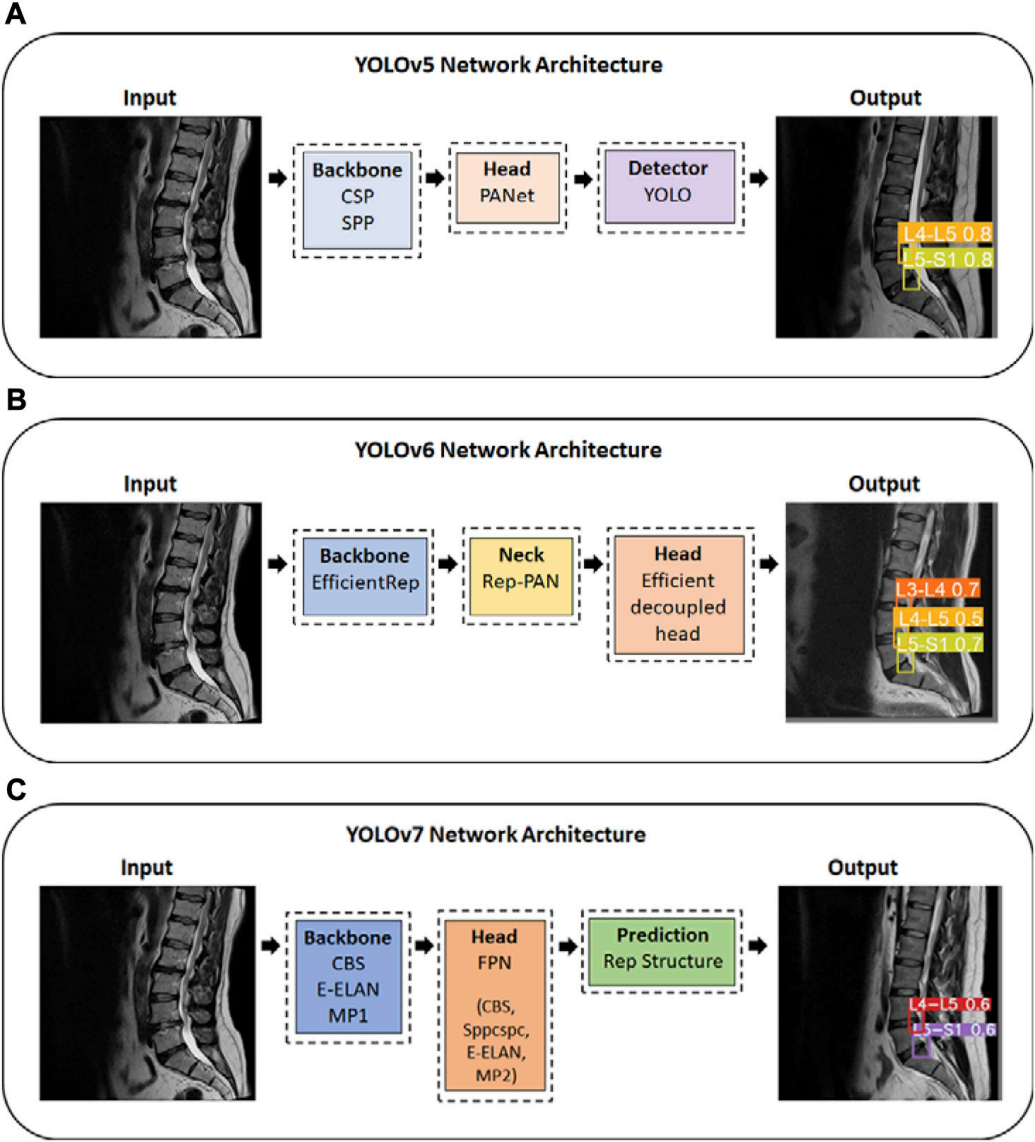
YOLO series Network Architecture; **(A)** YOLOv5 Network Architecture; **(B)** YOLOv6 Network Architecture; **(C)** YOLOv7 Network Architecture. YOLO, You Only Look Once. CSP, Cross Stage Partial. SPP, Spatial Pyramid Pooling. Rep, reparameterized. CBS, Convolutional, Batch normalization, SiLu activation blocks; E-ELAN, Extended efficient layer aggregation network; MP1/MP2, Max Pool-1/Max Pool-2; Sppcspc, Spatial Pyramid Pooling and Convolutional Spatial Pyramid Pool structure.

YOLOv5 uses an adaptive anchor strategy known as the auto anchor, in which the backbone comprises a focused structure and a CSP backbone. As a pre-training tool, auto anchor checks and adjusts anchor boxes if their fit is not optimal for the dataset and training settings. YOLOv5 network also uses a PANet neck to improve localization within layers (Jocher, 2020) (Figure 2A). Our study uses the YOLOv5x model, which is ideal for datasets containing smaller objects and is designed to provide high performance. According to a test using the MS COCO dataset test-dev 2017, YOLOv5x achieved an average percentage of 50.7% with an image size of 640 pixels and 200 frames per second (FPS) speed using an NVIDIA V100 (Terven and Cordova-Esparza, 2023). In YOLOv6, there are two scaled re-parameterizable backbones and necks to accommodate models of different sizes and a decoupled head efficiently implemented with a hybrid channel method. The hybrid channel has both single and multiple channels with enhanced quantization techniques that employ post-training quantization and channel-wise distillation. This has resulted in faster and more accurate detectors than previous versions of YOLOv5 (Li et al., 2022; Terven and Cordova-Esparza, 2023) (Figure 2B). According to a test using the MS COCO dataset test-dev 2017, the largest model of YOLOv6 achieved an average percentage of 57.2% with a speed of 29 FPS using an NVIDIA Tesla T4. Yolov7 proposed several architecture changes and a number of “bag-of-freebies,” which significantly increased the model’s accuracy without affecting its inference speed (Terven and Cordova-Esparza, 2023). YOLOv7 uses the extended efficient layer aggregation network (E-ELAN) backbone, model scaling, and model re-parameterization. The E-ELAN combines the characteristics of different groups by shuffling and merging cardinality in order to enhance the network’s learning capability without destroying the gradient path. The target detector in YOLOv7 is also implemented with extend and compound scaling, resulting in a substantial acceleration in detection (Wang et al., 2022) (Figure 2C). According to a test using the MS COCO dataset test-dev 2017, YOLOv7-E6 achieved an average percentage of 55.9% and AP_50_ of 73.5% with an image size of 1,280 pixels and 50 FPS on an NVIDIA V100 (Terven and Cordova-Esparza, 2023).

**FIGURE 3:**
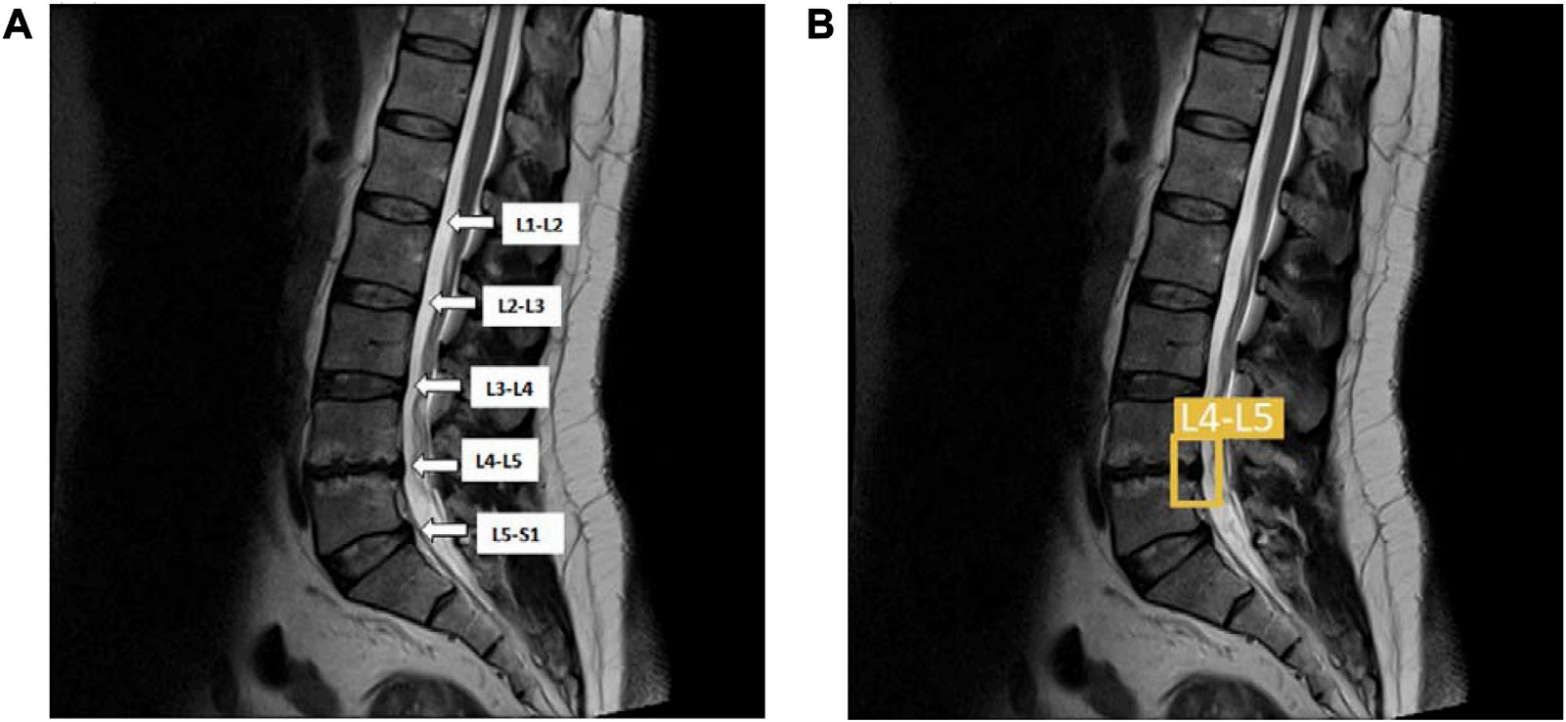
**(A)** The lumbar vertebrae and disc sections are from L1-L2, L2-L3, L3-L4, L4-L5, and L5-S1; **(B)** The same lumbar MRI displays LDH detection at L4-L5 and L5–S1. L1, first lumbar vertebra; L2, second lumbar vertebra; L3, third lumbar vertebra; L4, fourth lumbar vertebra; L5, fifth lumbar vertebra; S1, first sacral vertebra; LDH, lumbar disc herniation.

We used boundary box labeling in Roboflow to detect LDH of the lumbar intervertebral disc in 5 regions, the first and second lumbar vertebrae (L1-L2), the second and third lumbar vertebrae (L2-L3), third and fourth lumbar vertebrae (L3-L4), fourth and fifth lumbar vertebrae (L4-L5), the fifth lumbar vertebrae and the first sacral vertebrae (L5-S1). We put the LDH location labels on both the training and validation dataset. The lumbar vertebrae and disc sections are identified on the MRI scans, namely, L1-L2, L2-L3, L3-L4, L4-L5, and L5-S1 (Figure 3). YOLOv5x, YOLOv6, and YOLOv7 were trained to locate the LDH region on MRI images. This study was conducted using Windows 10 running Python 3.7.6 on a machine with the following specifications: Core (TM) i7-11700 CPU, 32 GB RAM, and an NVIDIA GeForce RTX 3090 GPU with 24 GB of GDDR6X memory. In this study, we trained the annotated dataset of YOLOv5, YOLOv6, and YOLOv7 using 16 batch sizes and 100 epochs.

### 2.3 Images augmentation

The dataset’s insufficient quantity of images can cause overfitting or underfitting and is one of many factors that affect deep learning performance. Earlier studies have used data augmentation to prevent and mitigate overfitting for deep learning (Ciregan et al., 2012; Abdelhafiz et al., 2019). During training, the augmentation addressed the issue of an excessively homogeneous dataset and improved the performance of deep learning models by simulating real-world situations.

**FIGURE 4:**
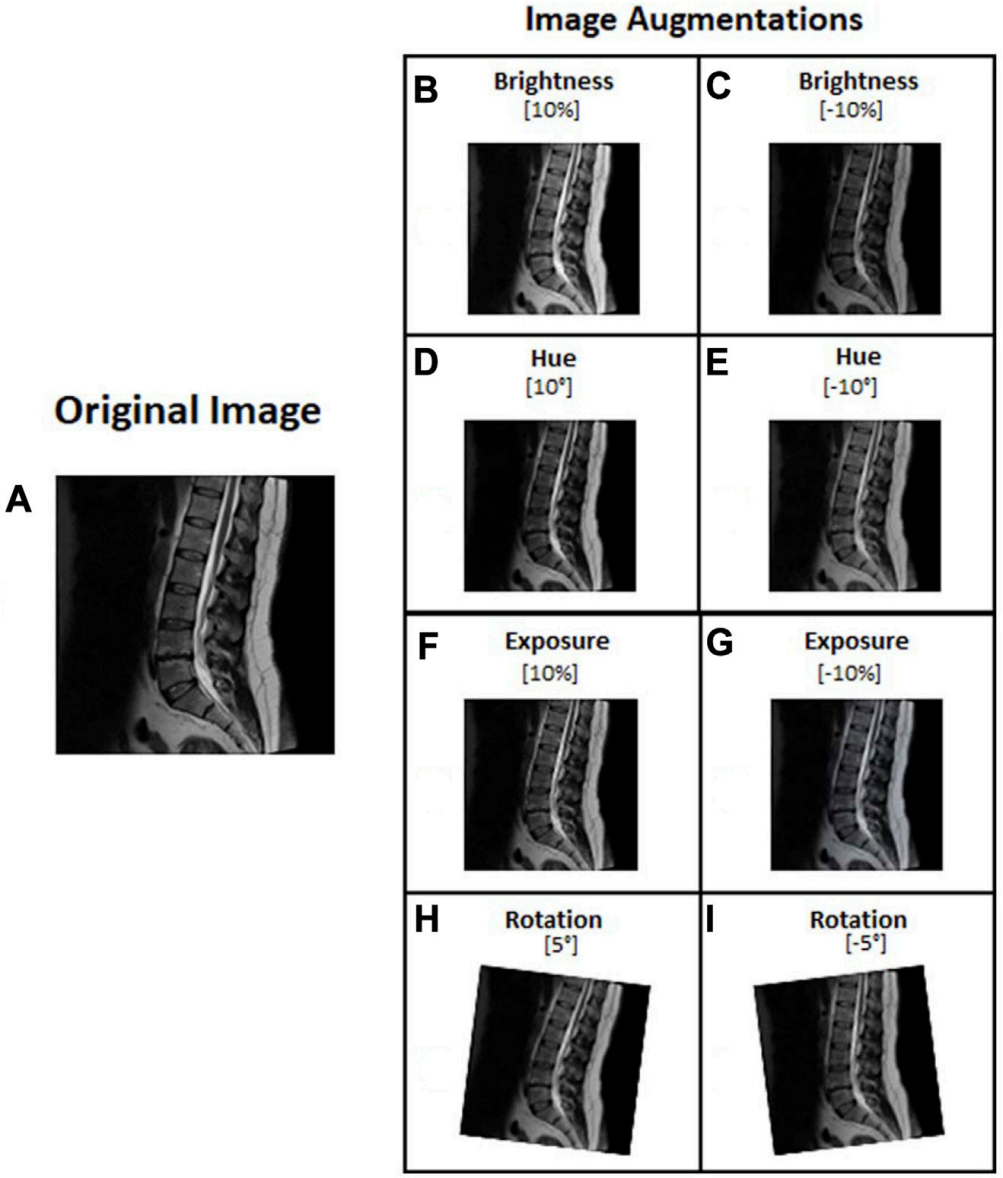
Lumbar images augmentation: **(A)** Original image, **(B)** Brightness 10%, **(C)** Brightness −10%, **(D)** Hue 10°, **(E)** Hue −10°, **(F)** Exposure 10%, **(G)** Exposure −10%, **(H)** rotation 5°, and **(I)** rotation −5°.

In image processing, the augmentation type and range settings are used to increase the volume and features of the image (Dao, 2019; Sánchez-Peralta et al., 2020). Our study selected the augmentation types in brightness, hue, exposure, and rotation to achieve the best results (Hussain, 2017; Sánchez-Peralta et al., 2020). We augmented the images with Roboflow after we had finished labeling them. The brightness, hue, and exposure adjustments represent various magnetic resonance machines and room lighting. The image rotation feature simulates various patient positions during MRI capture. Configuration images for YOLOv5, YOLOv6, and YOLOv7 were set to the brightness 10% and −10%, hue 10° and −10°, exposure 10% and −10%, and rotation 5° and −5° (Figure 4).

### 2.4 Deep learning performance

In most cases, The YOLO algorithm predicts the bounding box of the training and validation of the YOLO results using the average precision (AP) and the mean average precision (mAP) parameters on the LDH regions from L1-L2, L2-L3, L3-L4, L4-L5, and L5-S1 (Tsai et al., 2021). AP can be used as a comprehensive evaluation index to balance the effects of Precision and Recall. And we use a simple average F1 score and the AP value as an additional measure to demonstrate how well the methods perform on a complete dataset (Haque and Neubert, 2020). We selected the following metrics to assess the algorithm’s performance: Precision, Recall, AP, mAP, and F1 score. We then compared the Precision, Recall, AP, mAP, and F1 score for all LDH regions of YOLOv5x, YOLOv6, and YOLOv7 from L1-L2, L2-L3, L3-L4, L4-L5, and L5-S1, to determine which model was most suitable. A larger performance value indicates a more accurate model (Loram et al., 2020). It is also essential to use the mAP index to determine the network model’s overall performance as well as to prevent extreme and weak functioning during the evaluation process. This study further calculates and validates the performance of YOLOv5x, YOLOv6, and YOLOv7 to detect LDH. The three models were evaluated using the Accuracy metric.

The formula of the Precision (Eq. 1), Recall (Eq. 2), AP (Eq. 3), mAP (Eq. 4), F1 score (Eq. 5), Accuracy (Eq. 6) are as follows:

$$Precision=\frac{Correctly\;Predicted\;Disease\;Pixels}{Total\;Number\;of\;Predicted\;Disease\;Pixels}=\frac{TP}{TP+FP}$$ (1)

Precision, as defined above, measures the percentage of correctly predicted disease pixels corresponding to the ground truth. Precision is an important performance measure since it is sensitive to over-segmentation, leading to low precision scores [29]. TP, True Positive; FP, False Positive.

$$Recall=\frac{Correctly\;Predicted\;Disease\;Pixels}{Total\;Number\;of\;Ground\;Truth}=\frac{TP}{TP+FN}$$ (2)

Recall, as defined above, is an indicator of the proportion of correctly predicted disease pixels corresponding to the ground truth. It is susceptible to under-segmentation, resulting in low recall scores (Haque and Neubert, 2020). TP, True Positive; FN, False Negative.

$$AP=\sum_{n}\left(R_{n}-R_{n-1}\right)P_{n}$$ (3)

AP is the area under the precision-recall curve. It can be used as a comprehensive evaluation index to balance the effects of Precision and Recall. AP is calculated for each class separately (Tsai et al., 2021). AP; average precision; R, Recall; P, Precision; n, threshold number.

$$mAP=\frac{1}{N}\sum_{i=1}^{N}APi$$ (4)

The mAP is the average of AP over all detected classes and is used to evaluate the training, validate the results, and determine the overall model performance (Tsai et al., 2021). N, total number of the class; i, a score function to show an object similarity.

$$F1\;score=2\frac{Precision\times Recall}{Precision+Recall}$$ (5)

F1 score, is a weighted average of Precision versus Recall. One point is added to Precision if the result is relevant, and one point is added to Recall if at least one result is relevant. This way, a model’s performance can be measured effectively (Tsai et al., 2021).

$$Accuracy=\frac{TP+TN}{TP+TN+FP+FN}$$ (6)

Accuracy, as defined above, is calculated by taking the number of correctly predicted samples out of all possible samples. TP, True Positive; TN, True Negative; FP, False Positive; FN, False Negative.

## 3 Results

This study examined the performance of YOLOv5x, YOLOv6, and YOLOv7 in non-augmented (non-AUG) and augmented (AUG) dataset, compared the overall performance and the detection of regions on L1-L2, L2-L3, L3-L4, L4-L5, and L5-S1, and observed the training durations of YOLOv5x, YOLOv6, and YOLOv7.

### 3.1 Performance of the non-AUG and AUG dataset

The dataset includes 550 trained images without augmentation (550-non-AUG) and 550 trained images with 3 times augmentation (550-AUG). We compared the increased performance rates of the 550-non-AUG dataset to the 550-AUG dataset of all YOLO models by evaluating the increment rate of the mAP, which we calculated manually. The mAP is calculated based on Precision and Recall and thus can be used to determine the better model between the non-AUG and AUG YOLO models. There was a 0.34% increment rate between YOLOv5x and YOLOv5x-AUG, a 12.35% increment rate between YOLOv6 and YOLOv6-AUG, and a 1.51% increment rate between YOLOv7 and YOLOv7-AUG. While YOLOv6-AUG has the highest increment, its mAP performance remains slightly lower than YOLOv5x-AUG, with 2% less performance. Based on the metrics performance evaluation, the 550-AUG dataset outperforms the 550-non-AUG dataset on all YOLO models (Table 1).

**TABLE 1:** YOLOv5x, YOLOv6, YOLOv7, metrics performance comparison in the 550-trained dataset.

Metrics performance comparison	Model performance evaluation in the 550-trained dataset					
	YOLOv5x		YOLOv6		YOLOv7	
	Non-AUG (%)	AUG (%)	Non-AUG (%)	AUG (%)	Non-AUG (%)	AUG (%)
Precision	82.20	75.00	62.60	90.70	44.10	46.30
Recall	84.40	90.00	86.00	72.00	75.80	68.40
F1 score	83.29	81.82	72.50	80.30	55.76	55.22
mAP	89.00	89.30	77.70	87.30	59.70	60.60
AP (L1-L2)	69.30	82.80	65.10	79.30	37.40	24.50
AP (L2-L3)	90.20	84.10	58.70	73.60	37.90	50.00
AP (L3-L4)	93.60	94.70	84.70	95.80	49.20	50.20
AP (L4-L5)	96.80	97.30	97.30	97.80	93.50	93.00
AP (L5-S1)	94.90	87.60	82.80	90.20	80.30	85.20

Note: YOLO, you only look once; AUG, with augmentation; non-AUG, without augmentation; mAP, mean average precision; AP, average precision; L1, first lumbar vertebra; L2, second lumbar vertebra; L3, third lumbar vertebra; L4, fourth lumbar vertebra; L5, fifth lumbar vertebra; S1, first sacral vertebra.

### 3.2 Performance of deep learning model

**FIGURE 5:**
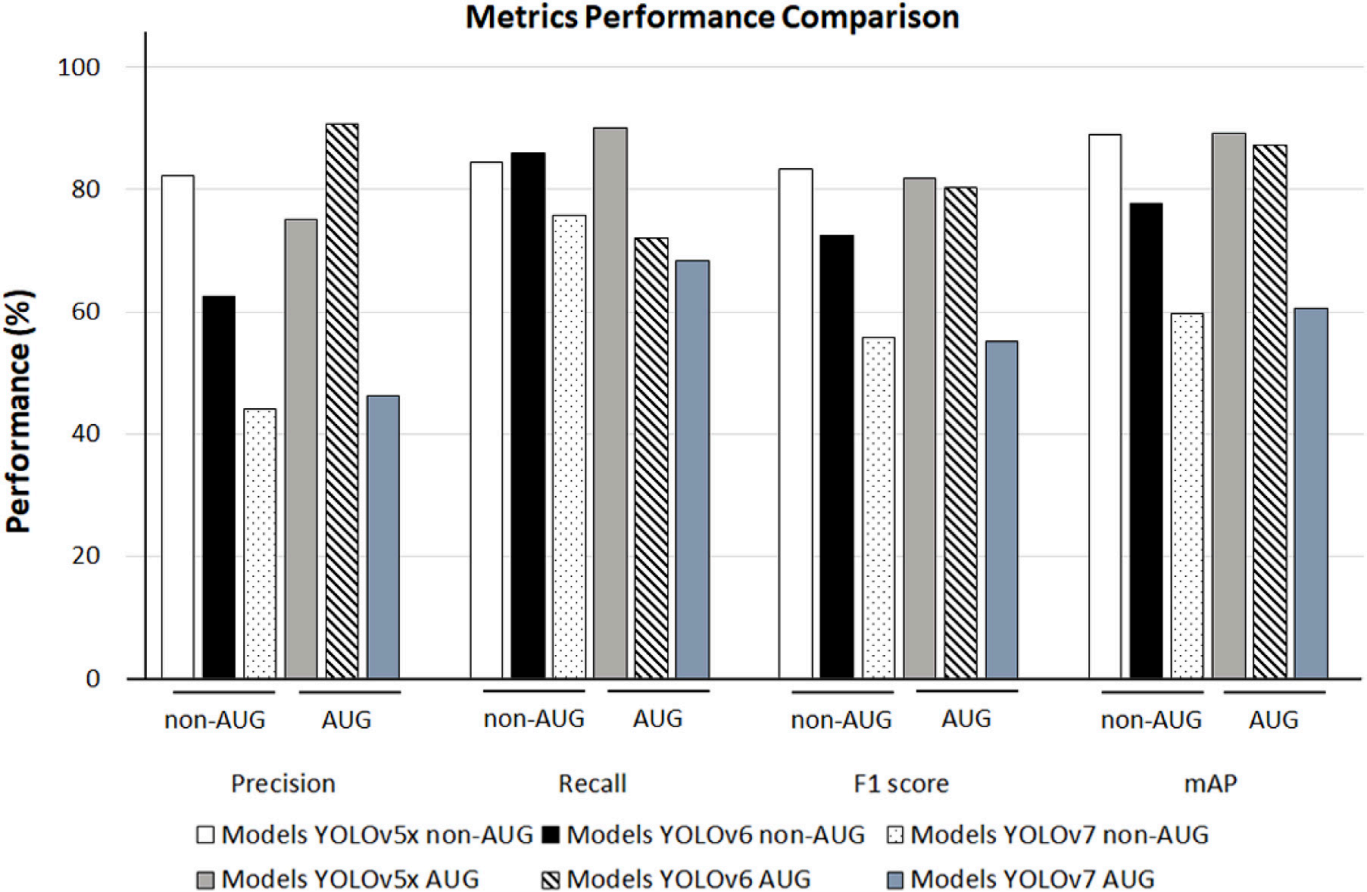
The 550-non-AUG and 550-AUG metric performance comparison of Precision, Recall, F1 score, mAP using YOLOv5x, YOLOv6, and YOLOv7. YOLO, You Only Look Once; AUG, with augmentation; non-AUG, without augmentation; mAP, mean average precision.

When comparing the model performance in Table 1, YOLOv5x in 550-AUG dataset has the highest performance value amongst all the YOLO models tested. Hence, we further compared the performance of YOLO models to other deep learning models in 550-AUG. The results revealed that YOLOv5x showed the highest Recall, F1-score, and mAP compared to all YOLO models, with 90.00%, 81.82%, and 89.30%, respectively. YOLOv5x only fell short in the Precision compared to YOLOv6, with 75% for YOLOv5x and 90.70% for YOLOv6. We then examined the mAP difference to evaluate the overall model performance on all models. YOLOv5x showed the highest mAP at 89.30%, YOLOv6 showed a lower mAP at 87.30%, and YOLOv7 showed the lowest mAP at 60.60% (Figure 5).

### 3.3 Performance of region detection

**FIGURE 6:**
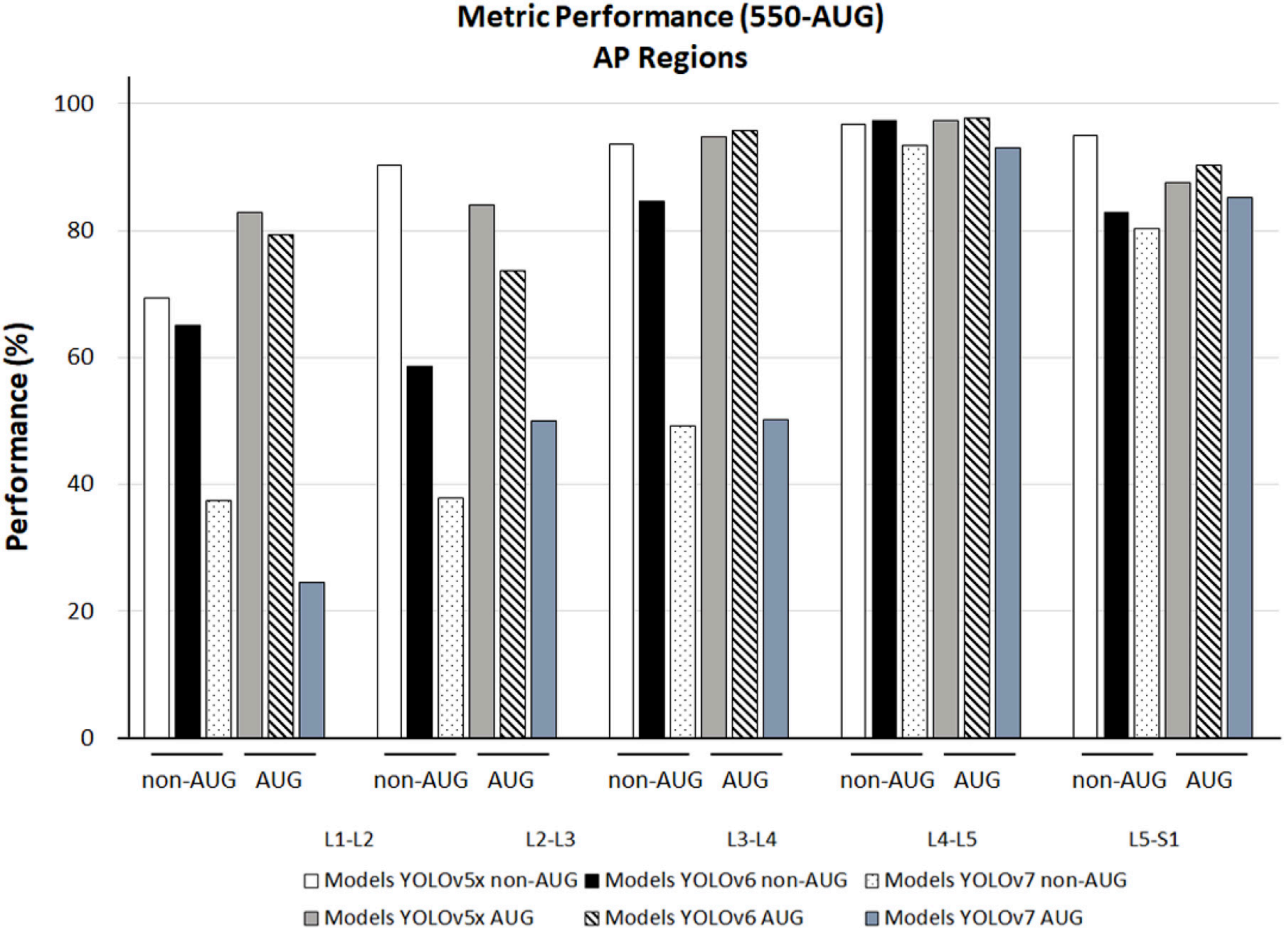
The 550-non-AUG and 550-AUG metrics performance comparison of L1-L2, L2-L3, L3-L4, L4-L5, and L5-S1 using YOLOv5x, YOLOv6, and YOLOv7. YOLO, You Only Look Once; AUG, with augmentation; non-AUG, without augmentation; AP, average precision; L1, first lumbar vertebra; L2, second lumbar vertebra; L3, third lumbar vertebra; L4, fourth lumbar vertebra; L5, fifth lumbar vertebra; S1, first sacral vertebra.

We observed the different AP outcomes in each lumbar region on all the models in the non-AUG and AUG dataset. YOLOv5x non-AUG showed AP values above 90% in 4 regions, L2-L3, L3-L4, L4-L5, and L5-S1 at 90.20%, 93.60%, 96.80%, and 94.90%, respectively, except in L1-L2, which is 69.30%. YOLOv6 showed the highest AP amongst all YOLO models tested in L3-L4, L4-L5, and L5-S1 at 95.80%, 97.80%, and 90.20%, respectively. Amongst all YOLO models during training, YOLOv7 showed the lowest performance on all L1-S1 regions (Figure 6).

Furthermore, we also calculated and validated the accuracy performance of YOLOv5x, YOLOv6, and YOLOv7 models. The YOLOv5x and YOLOv7 in non-AUG and AUG dataset showed accuracy above 70% in all L1-S1 lumbar regions. In comparison, YOLOv6 in the AUG dataset showed lower accuracy with a value of 69.77% in L3-L4, and in the non-AUG dataset showed the lowest accuracy with values of 69.77%, 67.24%, 63.60%, and 67.75% in L1-L2, L2-L3, L3-L4, and L4-L5 respectively (Table 2).

**TABLE 2:** Comparison of lumbar disc region detection accuracy.

Models	Dataset	Lumbar disc region detection accuracy				
		L1-L2 (%)	L2-L3 (%)	L3-L4 (%)	L4-L5 (%)	L5-S1 (%)
YOLOv5x	non-AUG	77.00	72.00	70.00	73.00	72.00
YOLOv5x	AUG	75.75	70.69	72.00	72.81	70.48
YOLOv6	non-AUG	69.77	67.24	63.60	67.75	74.04
YOLOv6	AUG	74.93	70.77	69.77	72.63	72.18
YOLOv7	non-AUG	76.21	75.92	72.52	71.61	70.14
YOLOv7	AUG	73.95	71.05	71.33	70.88	72.34

Note: YOLO, you only look once; AUG, with augmentation; non-AUG, without augmentation; L1, first lumbar vertebra; L2, second lumbar vertebra; L3, third lumbar vertebra; L4, fourth lumbar vertebra; L5, fifth lumbar vertebra; S1, first sacral vertebra.

### 3.4 Performance in training duration and weight

We also observed the performance in training duration and the weight difference on all YOLO models according to non-AUG and AUG dataset. YOLOv5x non-AUG showed the shortest duration and the lightest folder weight after training, with 0.097 h and 14.8 MB, respectively. YOLOv5x AUG showed a slightly longer training duration and weight than YOLOv5x non-AUG by 3 times value, but the weight after training was similar. YOLOv6 non-AUG showed a slightly longer duration and heavier weight than YOLOv5x non-AUG with 0.140 h and 264 MB, respectively. However, YOLOv6 AUG showed a shorter duration than YOLOv5x AUG, although the weight is still far heavier than YOLOv5x AUG by 16 times value. And, YOLOv7 non-AUG and YOLOv7 AUG showed the longest duration and heaviest folder weight after training compared to all YOLOv5x and YOLOv6 models (Table 3).

**TABLE 3:** The 550-non-AUG and 550-AUG training duration and weight using YOLOv5x, YOLOv6, and YOLOv7.

Models	Dataset	Epoch	Batch size	Total image	Training duration (hours)	Weight (MB)
YOLOv5x	non-AUG	100	16	550	0.097	14.8
YOLOv5x	AUG	100	16	1,650	0.274	14.8
YOLOv6	non-AUG	100	16	550	0.140	264.0
YOLOv6	AUG	100	16	1,650	0.258	239.0
YOLOv7	non-AUG	100	16	550	0.361	5,290.0
YOLOv7	AUG	100	16	1,650	0.959	5,290.0

Note: YOLO, you only look once; AUG, with augmentation; non-AUG, without augmentation.

## 4 Discussion

We demonstrated the possibility of using the YOLOv5x, YOLOv6, and YOLOv7 to determine the LDH in MRI images based on bulging, protrusion, extrusion, and sequestration. In this study, YOLOv5x AUG was considered the most suitable model among the other YOLO models tested, showing the highest mAP performance. Moreover, our study showed that YOLOv5x non-AUG was an efficient LDH region predictor, as it had the highest AP scores in 4 regions, L2-L3, L3-L4, L4-L5, and L5-S1. YOLOv5x also showed the shortest training duration and the lightest weight compared to all YOLO models tested.

Based on the results from the current study, the YOLOv5x AUG had the best mAP performance among all YOLO models trained. YOLOv5x network architecture with PANet neck combines the 550-AUG images before they are sent for prediction, increasing the accuracy (Jocher, 2020). During training, YOLO models provide several parameters such as Precision, Recall, F1-Score, mAP, AP per lumbar disc region (L1-S1). The Precision and Recall are parameters that indicated the data sensitivity and predicted pixels accordingly, which are not enough to determine the overall performance of a deep learning model. The efficiency balance performance measured by F1-score and overall performance measured by mAP is calculated based on Precision and Recall to determine the best model among YOLOv5x, YOLOv6, and YOLOv7. From Table 1, YOLOv6-AUG has the highest detection region in L2-L3, L4-L5, and L5-S1. However, the overall performance of YOLOv6-AUG measured by mAP is still slightly lower than YOLOv5x-AUG. Thus based on the results of this study, YOLOv5x demonstrated a promising performance as a deep learning model for identifying LDH in MRI compared to all models trained. The greater mAP value observed with YOLOv5x proved the model is more accurate in predicting the presence of LDH in the MRI, indicating its potential for clinical use. Although YOLOv6 and YOLOv7 are the latest versions of YOLO, their effectiveness in biomedical applications is not substantially better. Our results are consistent with a study that found YOLOv6 and YOLOv7 to be less effective at detecting biomedical images than YOLOv5, possibly as a result of preliminary experiments, tune-ups, and revisions since these versions were published recently (Chen et al., 2022).

Our study found that the 550-AUG dataset had an improved performance compared to the 550 non-AUG dataset. However, other parameters in AUG dataset results, such as Precision, and F1 score of YOLOv5x, and Recall and F1 score of YOLOv7, were less accurate than the non-AUG dataset, as shown in Table 1. In this study, augmentation is performed using Roboflow, which randomly places the augmentation following the parameters we specify. However, a problem with random augmentation is that each image will have different combinations assigned, and not every augmentation will be applied to every image (Iwana and Uchida, 2021). It is possible for some images to be assigned a brightness and rotation augmentation, while others might be assigned a hue and exposure augmentation or a combination of these four augmentations. In general, the results of the augmentation were mixed. Several data augmentation methods have improved the accuracy, but some combinations with rotation methods might be detrimental (Iwana and Uchida, 2021). Based on our result in Table 1, we demonstrated the efficiency of training YOLOv5x model using a non-AUG dataset.

In the lumbar regions, our training result showed that YOLOv5x non-AUG had a more stable performance compared to all models, as it had detection performance above 90% in 4 regions, such as L2-L3, L3-L4, L4-L5, and L5-S1. In these regions, the disc herniation might cause radicular pain, which may compress the nerve root, resulting in pain and dysfunction symptoms (Reihani-Kermani, 2003; Fang et al., 2016). Deep learning’s ability to recognize small objects, such as bulging, protrusion, extrusion, and sequestration in MRI lumbar, helps identify the LDH on different sizes and scales (Liu et al., 2020). During training, YOLOv5x non-AUG only demonstrated slightly low detection in L1-L2, which is not in the lower region where disc disease is commonly found (Katz et al., 2022). However, we validate the accuracy performance of YOLOv5x non-AUG and further confirm the potential to use the non-AUG dataset for LDH detection with all regions detection above 70%. Therefore, deep learning utilization may contribute to the early identification of spine abnormalities with greater accuracy in the four lower regions: L2-L3, L3-L4, L4-L5, and L5-S1, thereby helping clinicians determine the appropriate therapy in the earliest possible time and protecting patients from harmful consequences.

Deep learning models can be challenging and expensive to train, taking hours or weeks to complete (Lee et al., 2017). Our results showed that YOLOv5x had the shortest training duration and the lightest weight, showing that this model is most efficient in detecting LDH. To the best of our knowledge, this is the first study to compare the training duration of LDH detection across YOLOv5x, YOLOv6, and YOLOv7 models. A model’s performance during training will determine how well it is able to perform when a user eventually uses it. Clinical settings could certainly benefit from using this as a computational reference in the future.

This study compared related studies to evaluate the LDH detection performance and total dataset used in MRI based on other models’ performances (Table 4). Alomari et al. (2014) showed a high accuracy detection using four classification stages and detection in a two-stage detector model to identify LDH). Wang et al., and Su et al., also showed a high accuracy detection using a two-stage detector model to identify LDH (Wang et al., 2020; Su et al., 2022). Compared with other studies, (Zhou et al., 2019) used 2,739 images; (Su et al., 2022) used 15,254 and 1,273 images; and (Guinebert et al., 2022) used 40 volumes of MRI (3,660 images) to train for deep learning. Their dataset included more than a thousand images. This study used 550 (non-AUG) and 1,650 (AUG) lumbar MRI images to train YOLOv5x, YOLOv6, and YOLOv7. Our previous study showed a higher accuracy with YOLOv3 than our current study, but it used twice as many slices to test the deep learning (Tsai et al., 2021). As our current dataset contains more patients, we will see a greater variation of LDH in MRI during the training process, which produces a reasonable result with a similar small-scale dataset. Compared to other studies, these results demonstrate the competitiveness of non-AUG dataset. In addition, we also used a single-stage detector, whereas some of the others used a two-stage detector model.

**TABLE 4:** The comparison of LDH detection performance with the related literature study using lumbar MRI with different deep learning algorithms.

References	Method	Precision	Recall	F1-score	mAP	Accuracy	Total images	No. of patients	No. of slices
Alomari et al. (2014)	ASM + GVF-Snake	—	—	—	—	93.90%	390	65	6
Zhou et al. (2019)	Siamese Network	98.90%	—	—	—	98.60%	2,739	2,739	1
Wang et al. (2020)	DenseNet + Inception-Resnet-V2	96.65%	95.13%	—	—	96.22%	790	395	2
Tsai et al. (2021)	YOLOv3 (AUG)	87.20%	91.70%	89.40%	92.40%	81.10%	714	65	11
Su et al. (2022)	ResNet-50 + 3 FC networks (1)	—	—	—	—	84.17%	15,254	1,115	—
	ResNet-50 + 3 FC networks (2)	—	—	—	—	74.20%	1,273	100	—
Guinebert et al. (2022)	YOLOv5x	75.00%	76.50%	—	—	—	3,660	244	15
Our study	YOLOv5x	82.20%	84.40%	83.29%	89.00%	72.80%	550	110	5
	YOLOv6	62.60%	86.00%	72.50%	77.70%	68.48%	550	110	5
	YOLOv7	44.10%	75.80%	55.76%	59.70%	73.28%	550	110	5
	YOLOv5x (AUG)	75.00%	90.00%	81.82%	89.30%	72.35%	1,650	110	5
	YOLOv6 (AUG)	90.70%	72.00%	80.30%	87.30%	72.06%	1,650	110	5
	YOLOv7 (AUG)	46.30%	68.40%	55.22%	60.60%	71.91%	1,650	110	5

In recent years, clinical practice has significantly changed due to the use of deep learning models in diagnosis assistance, including the automatic detection of LDH (Lee and Yoon, 2021). In medical imaging, deep learning can provide efficient and accurate results and is regarded as one of the most promising methods for future application in the healthcare sector (Razzak et al., 2018). To prevent misdiagnosis, patients with borderline LDH visibility may require multiple MRI, which can be very costly (Bruno et al., 2015). However, even in the presence of blurriness or noise, deep learning models can be trained to be more robust at identifying LDH (Guinebert et al., 2022). Consequently, these deep learning models could provide clinicians with a helpful LDH prediction for more accurate diagnosis, thus reducing hospital visits and optimizing healthcare costs.

**FIGURE 7:**
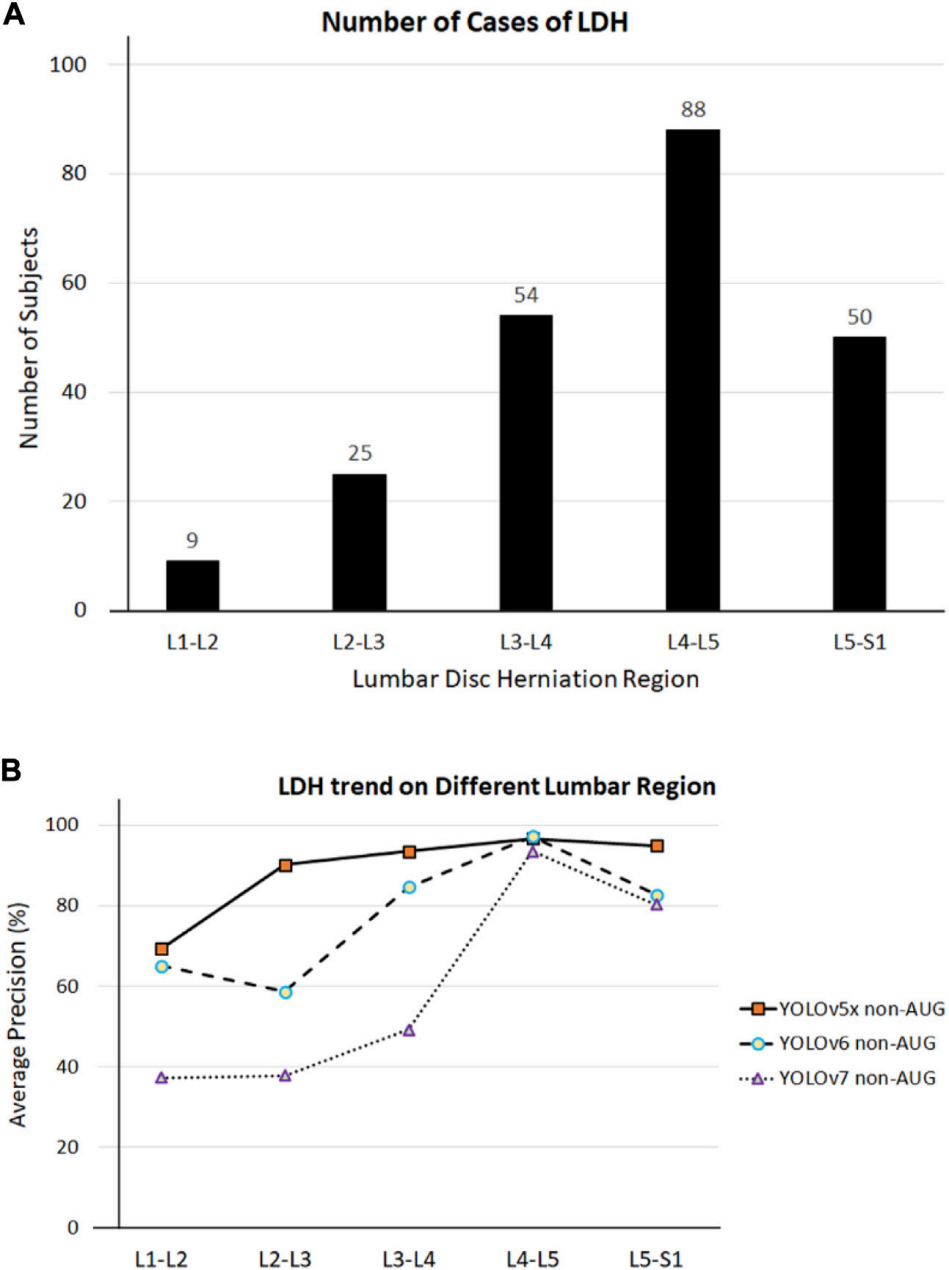
**(A)** LDH cases in different lumbar vertebrae regions. **(B)** LDH cases and average precision (AP) related trend in different lumbar vertebrae regions. L1, first lumbar vertebra; L2, second lumbar vertebra; L3, third lumbar vertebra; L4, fourth lumbar vertebra; L5, fifth lumbar vertebra; S1, first sacral vertebra.

Our first limitation is the lack of AP value of the L1-L2 region during training using our most efficient model YOLOv5x in the non-AUG dataset. Such an issue might be because the LDH development in MRI may not be visible in these regions, or the protrusion may be too small to be noticed. In addition, the number of subjects with LDH in L1-L2 regions from the dataset was also limited. There were 226 LDH cases separated from L1 to S1 at 9, 25, 54, 88, and 50 (Figure 7A). Based on the number of cases, our study was also consistent with the findings of other studies about LDH (Faur et al., 2019; Tsai et al., 2021). L1-L2 had the lowest frequency of LDH, while L4-L5 had the highest frequency of symptoms. As a whole, the AP value for L4-L5 was more distinguishable than the number of LDH for each of the lumbar vertebrae regions. The training results indicate that the number of symptoms affects deep learning in each LDH region (Figure 7B). Insufficient image numbers in the small dataset result in limited detection due to deep learning underfitting and overfitting.

Our second limitation is the LDH multi-labeling format on all YOLO models. In this study, we put the label only on the discs with bulges, protrusions, extrusions, and sequestrations as indicators of LDH in the MRI. Detailed labeling on each disc from L1-S1 to confirm the appearance of LDH might provide more specificity on the clinical diagnosis. However, putting confirmation labels on each disc in MRI might be challenging, as different regions will be affected by LDH. A multi-label classification typically requires additional effort in extracting and describing the associated label information to achieve satisfactory training results (Rastogi and Kumar, 2023). Furthermore, more labels require a larger dataset to avoid missing labels that could hinder the deep learning process. If incomplete labeled data is used for training, it may result in noisy classifiers with inadequate prediction capabilities (Wu et al., 2014).

As of today, YOLO is one of the fastest-growing and best algorithms available, with the current YOLOv8 algorithm being released in 2023. Our initial speculation by utilizing detection feature improvements in YOLOv8 may increase the accuracy of the LDH detection. Due to the speed, accuracy, and ease of use of YOLOv8, it is an excellent choice for several object detection, instance segmentation, and image classification applications (Ju and Cai, 2023). Despite this, Ju and Cai (2023) found that when they trained YOLOv8 on biomedical images, the accuracy range of the system was still 60%, which is still considered to be low in terms of detection accuracy. Because of these reasons, it is still uncertain whether YOLOv8 is capable of detecting LDH. It would be worth investigating the accuracy of YOLOv8 to detect the LDH accuracy in the L1-L2 region and to also implement a more detailed multi-labeling image of the LDH region in the future.

Our third limitation is using the MRI sagittal view, which lacks LDH visualization of the specific annular tear angle. A deep learning algorithm can identify the annular tears in which the nucleus pulposus protrudes and compresses the lumbar discs (Su et al., 2022). From our sagittal view results, we were able to detect the LDH development based on the protrusion on the MRI image. A sagittal view is also used by neuroradiologists for the initial examination of the three lowest intervertebral discs—L3/L4, L4/L5, and L5/S1 (Katz et al., 2022). However, in most cases, neuroradiologists diagnose neural foraminal stenosis based on an axial view of the spine. Neural foraminal stenosis is important to indicate where an annular tear begins, which compresses the disc at a specific angle, either symmetrical or asymmetrical (Amin et al., 2017). Future studies might confirm the LDH development based on the annular tear on axial views. As such, we believe that deep learning detection does not replace medical personnel but provides fast access to additional information that accelerates initial diagnosis and helps focus on specific areas of concern. In this manner, the diagnosis may be made more quickly and with greater certainty before the team of multidisciplinary professionals decides on future surgery or treatment.

## 5 Conclusion

This study contributes to the automatic detection of LDH using deep learning and further identifies the best model for the YOLO series. Our study showed that YOLOv5x, YOLOv6, and YOLOv7 are promising deep learning methods for detecting LDH from MRI. In the present study, we observed that YOLOv5x AUG showed the highest overall performance based on mAP value. In addition, the YOLOv5x non-AUG showed stable LDH detection levels in L2-L3, L3-L4, L4-L5, and L5-S1 based on AP values which show competitiveness in using the non-AUG dataset for training. Further, YOLOv5x showed the most efficient training duration, which may prove useful in clinical settings where a computational application is required. Finally, this study demonstrated that YOLOv5x can detect LDH, and its application in biomedical imaging may be beneficial.

## Data Availability

The original contributions presented in the study are included in the article/Supplementary Material, further inquiries can be directed to the corresponding author.

## References

[B1] Abdelhafiz D. Yang C. Ammar R. Nabavi S. (2019). Deep convolutional neural networks for mammography: advances, challenges and applications. BMC Bioinforma. 20 (11), 281–320. 10.1186/s12859-019-2823-4 PMC6551243 31167642

[B2] Al-Kafri A. S. Sudirman S. Hussain A. Al-Jumeily D. Natalia F. Meidia H. (2019). Boundary delineation of MRI images for lumbar spinal stenosis detection through semantic segmentation using deep neural networks. IEEE Access 7, 43487–43501. 10.1109/access.2019.2908002

[B3] Alomari R. S. Corso J. J. Chaudhary V. Dhillon G. (2014). “Lumbar spine disc herniation diagnosis with a joint shape model,” in Computational methods and clinical applications for spine imaging (Springer), 87–98.

[B4] Amin R. M. Andrade N. S. Neuman B. J. (2017). Lumbar disc herniation. Curr. Rev. Musculoskelet. Med. 10 (4), 507–516. 10.1007/s12178-017-9441-4 28980275 PMC5685963

[B5] Azimi P. Yazdanian T. Benzel E. C. Aghaei H. N. Azhari S. Sadeghi S. (2020). A review on the use of artificial intelligence in spinal diseases. Asian Spine J. 14 (4), 543–571. 10.31616/asj.2020.0147 32326672 PMC7435304

[B6] Bruno M. A. Walker E. A. Abujudeh H. H. (2015). Understanding and confronting our mistakes: the epidemiology of error in radiology and strategies for error reduction. Radiographics 35 (6), 1668–1676. 10.1148/rg.2015150023 26466178

[B7] Chen E. Liao R. Shalaginov M. Y. Zeng T. H. (2022). “Real-time detection of acute lymphoblastic leukemia cells using deep learning,” in 2022 IEEE International Conference on Bioinformatics and Biomedicine (BIBM), Las Vegas, NV, USA, 6-8 Dec. 2022 (IEEE), 3788–3790. 10.1109/BIBM55620.2022.9995131

[B8] Chen K.-T. Tseng C. Sun L. W. Chang K. S. Chen C. M. (2021). Technical considerations of interlaminar approach for lumbar disc herniation. World Neurosurg. 145, 612–620. 10.1016/j.wneu.2020.06.211 32622922

[B9] Choi K.-C. Kim J. S. Lee D. C. Park C. K. (2017). Percutaneous endoscopic lumbar discectomy: minimally invasive technique for multiple episodes of lumbar disc herniation. BMC Musculoskelet. Disord. 18 (1), 329–336. 10.1186/s12891-017-1697-8 28764746 PMC5540429

[B10] Ciregan D. Meier U. Schmidhuber J. (2012). “Multi-column deep neural networks for image classification,” in 2012 IEEE conference on computer vision and pattern recognition, Hadhramout, Yemen, 15-16 Dec. 2019 (IEEE), 1–5. 10.1109/ICOICE48418.2019.9035162

[B11] Dao T. (2019). “A kernel theory of modern data augmentation,” in International conference on machine learning (PMLR). PMC6879382 31777848

[B12] Fang G. Zhou J. Liu Y. Sang H. Xu X. Ding Z. (2016). Which level is responsible for gluteal pain in lumbar disc hernia? BMC Musculoskelet. Disord. 17 (1), 356–364. 10.1186/s12891-016-1204-7 27550040 PMC4994246

[B13] Faur C. Patrascu J. M. Haragus H. Anglitoiu B. (2019). Correlation between multifidus fatty atrophy and lumbar disc degeneration in low back pain. BMC Musculoskelet. Disord. 20 (1), 414–416. 10.1186/s12891-019-2786-7 31488112 PMC6729014

[B14] Friska N. Sudirman S. (2021). Classification of sagittal lumbar spine MRI for lumbar spinal stenosis detection using transfer learning of a deep convolutional neural network. IEEE Explore.

[B15] Ghosh S. Chaudhary V. (2014). Supervised methods for detection and segmentation of tissues in clinical lumbar MRI. Comput. Med. Imaging Graph. 38 (7), 639–649. 10.1016/j.compmedimag.2014.03.005 24746606

[B16] Gopalakrishnan N. Nadhamuni K. Karthikeyan T. (2015). Categorization of pathology causing low back pain using magnetic resonance imaging (MRI). J. Clin. Diagnostic Res. JCDR 9 (1), TC17–TC20. 10.7860/JCDR/2015/10951.5470 PMC4347147 25738056

[B17] Guinebert S. Petit E. Bousson V. Bodard S. Amoretti N. Kastler B. (2022). Automatic semantic segmentation and detection of vertebras and intervertebral discs by neural networks. Comput. Methods Programs Biomed. Update 2, 100055. 10.1016/j.cmpbup.2022.100055

[B18] Haque I. R. I. Neubert J. (2020). Deep learning approaches to biomedical image segmentation. Inf. Med. Unlocked 18, 100297. 10.1016/j.imu.2020.100297

[B19] Hussain Z. (2017). “Differential data augmentation techniques for medical imaging classification tasks,” in AMIA annual symposium proceedings (American Medical Informatics Association). PMC5977656 29854165

[B20] Iwana B. K. Uchida S. (2021). An empirical survey of data augmentation for time series classification with neural networks. Plos one 16 (7), e0254841. 10.1371/journal.pone.0254841 34264999 PMC8282049

[B21] Jocher G. (2020). yolov5. Code repository.

[B22] Ju R.-Y. Cai W. (2023). Fracture detection in pediatric wrist trauma X-ray images using YOLOv8 algorithm. arXiv preprint arXiv:2304.05071. 10.1038/s41598-023-47460-7 PMC10654405 37973984

[B23] Katz J. N. Zimmerman Z. E. Mass H. Makhni M. C. (2022). Diagnosis and management of lumbar spinal stenosis: A review. Jama 327 (17), 1688–1699. 10.1001/jama.2022.5921 35503342

[B24] Lee D. Yoon S. N. (2021). Application of artificial intelligence-based technologies in the healthcare industry: opportunities and challenges. Int. J. Environ. Res. Public Health 18 (1), 271. 10.3390/ijerph18010271 33401373 PMC7795119

[B25] Lee J.-G. Jun S. Cho Y. W. Lee H. Kim G. B. Seo J. B. (2017). Deep learning in medical imaging: general overview. kjr 18 (4), 570–584. 10.3348/kjr.2017.18.4.570 PMC5447633 28670152

[B26] Li C. Li L. Jiang H. Weng K. Geng Y. Li L. (2022). YOLOv6: A single-stage object detection framework for industrial applications. arXiv preprint arXiv:2209.02976. 10.48550/arXiv.2209.02976

[B27] Liu Y. Hsu T. W. Chang C. Y. Liao W. H. Chang J. M. (2020). GODoc: high-throughput protein function prediction using novel k-nearest-neighbor and voting algorithms. World Sci. Res. J. 6 (11), 276–284. 10.1186/s12859-020-03556-9 PMC7672824 33203348

[B28] Loram I. Siddique A. Sanchez M. B. Harding P. Silverdale M. Kobylecki C. (2020). Objective analysis of neck muscle boundaries for cervical dystonia using ultrasound imaging and deep learning. IEEE J. Biomed. health Inf. 24 (4), 1016–1027. 10.1109/jbhi.2020.2964098 31940567

[B29] Martin B. I. Mirza S. K. Spina N. Spiker W. R. Lawrence B. Brodke D. S. (2019). Trends in lumbar fusion procedure rates and associated hospital costs for degenerative spinal diseases in the United States, 2004 to 2015. Spine 44 (5), 369–376. 10.1097/brs.0000000000002822 30074971

[B30] Mushtaq M. Akram M. U. Alghamdi N. S. Fatima J. Masood R. F. (2022). Localization and edge-based segmentation of lumbar spine vertebrae to identify the deformities using deep learning models. Sensors 22 (4), 1547. 10.3390/s22041547 35214448 PMC8879729

[B31] Rastogi R. Kumar S. (2023). Discriminatory label-specific weights for multi-label learning with missing labels. Neural Process. Lett. 55 (2), 1397–1431. 10.1007/s11063-022-10945-z

[B32] Razzak M. I. Naz S. Zaib A. (2018). “Deep learning for medical image processing: overview, challenges and the future,” in Classification in BioApps. Lecture notes in computational vision and Biomechanics (Springer, Cham, 26, 323–350. 10.1007/978-3-319-65981-7_12

[B33] Reihani-Kermani H. (2003). Level-diagnosis of lumbar disc herniation. 10.5144/0256-4947.2004.273 PMC6148114 15387493

[B34] Sánchez-Peralta L. F. Picón A. Sánchez-Margallo F. M. Pagador J. B. (2020). Unravelling the effect of data augmentation transformations in polyp segmentation. Int. J. Comput. assisted radiology Surg. 15 (12), 1975–1988. 10.1007/s11548-020-02262-4 PMC7671995 32989680

[B35] Su Z. H. Liu J. Yang M. S. Chen Z. Y. You K. Shen J. (2022). Automatic grading of disc herniation, central canal stenosis and nerve roots compression in lumbar magnetic resonance image diagnosis. Front. Endocrinol. (Lausanne) 13, 890371. 10.3389/fendo.2022.890371 35733770 PMC9207332

[B36] Sudirman S. Kafri A. A. Natalia F. Meidia H. Afriliana N. Al-Rashdan W. (2019a). Lumbar spine MRI dataset. Mendeley Data.

[B37] Sudirman S. Kafri A. A. Natalia F. Meidia H. Afriliana N. Al-Rashdan W. (2019b). MATLAB source code for developing ground truth dataset, semantic segmentation, and evaluation for the lumbar spine MRI dataset. Mendeley Data.

[B38] Terven J. Cordova-Esparza D. (2023). A comprehensive review of YOLO: From YOLOv1 to YOLOv8 and beyond. arXiv preprint arXiv:2304.00501.

[B39] Tsai J.-Y. Hung I. Y. J. Guo Y. L. Jan Y. K. Lin C. Y. Shih T. T. F. (2021). Lumbar disc herniation automatic detection in magnetic resonance imaging based on deep learning. Front. Bioeng. Biotechnol. 9, 691. 10.3389/fbioe.2021.708137 PMC8416668 34490222

[B40] Vialle L. R. Vialle E. N. Suárez Henao J. E. Giraldo G. (2010). Lumbar disc herniation. Rev. Bras. Ortop. (English Ed. 45 (1), 17–22. 10.1016/s2255-4971(15)30211-1 PMC4799068 27019834

[B41] Wang C.-Y. Bochkovskiy A. Liao H.-Y. M. (2022). YOLOv7: Trainable bag-of-freebies sets new state-of-the-art for real-time object detectors. arXiv preprint arXiv:2207.02696.

[B42] Wang Z. Qin J. Huang J. Wang Y. Li J. (2020). “Automatic diagnosis of disc herniation based on DenseNet fusion model,” in 2020 8th international conference on digital home (ICDH) (IEEE).

[B43] Wu B. Liu Z. Wang S. Hu B. G. Ji Q. (2014). “Multi-label learning with missing labels,” in 2014 22nd International Conference on Pattern Recognition, Stockholm, Sweden, 24-28 Aug. 2014 (IEEE), 1964–1968. 10.1109/ICPR.2014.343

[B44] Yung N. D. T. Wong W. K. Juwono F. H. Sim Z. A. (2022). “Safety helmet detection using deep learning: implementation and comparative study using YOLOv5, YOLOv6, and YOLOv7,” in 2022 International Conference on Green Energy, Computing and Sustainable Technology (GECOST), Miri Sarawak, Malaysia, 26-28 Oct. 2022 (IEEE), 164–170. 10.1109/GECOST55694.2022.10010490

[B45] Zhou Y. Liu Y. Chen Q. Gu G. Sui X. (2019). Automatic lumbar MRI detection and identification based on deep learning. J. digital imaging 32, 513–520. 10.1007/s10278-018-0130-7 PMC6499854 30338477

